# Economic Evaluation of Family Planning Interventions in Low and Middle Income Countries; A Systematic Review

**DOI:** 10.1371/journal.pone.0168447

**Published:** 2016-12-19

**Authors:** Neily Zakiyah, Antoinette D. I. van Asselt, Frank Roijmans, Maarten J. Postma

**Affiliations:** 1 Unit of PharmacoTherapy, -Epidemiology & -Economics (PTEE), Department of Pharmacy, University of Groningen, Groningen, The Netherlands; 2 Unit of Patient Centered Health Technology Assessment, Department of Epidemiology, University Medical Center Groningen, University of Groningen, Groningen, The Netherlands; 3 Unit Training, Consultancy and Projects, i+Solutions, Woerden, The Netherlands; 4 Institute of Science in Healthy Aging & healthcaRE (SHARE), University Medical Center Groningen, University of Groningen, Groningen, The Netherlands; London School of Economics and Political Science, UNITED KINGDOM

## Abstract

**Background:**

A significant number of women in low and middle income countries (L-MICs) who need any family planning, experience a lack in access to modern effective methods. This study was conducted to review potential cost effectiveness of scaling up family planning interventions in these regions from the published literatures and assess their implication for policy and future research.

**Study design:**

A systematic review was performed in several electronic databases i.e Medline (Pubmed), Embase, Popline, The National Bureau of Economic Research (NBER), EBSCOHost, and The Cochrane Library. Articles reporting full economic evaluations of strategies to improve family planning interventions in one or more L-MICs, published between 1995 until 2015 were eligible for inclusion. Data was synthesized and analyzed using a narrative approach and the reporting quality of the included studies was assessed using the Consolidated Health Economic Evaluation Reporting Standards (CHEERS) statement.

**Results:**

From 920 references screened, 9 studies were eligible for inclusion. Six references assessed cost effectiveness of improving family planning interventions in one or more L-MICs, while the rest assessed costs and consequences of integrating family planning and HIV services, concerning sub-Saharan Africa. Assembled evidence suggested that improving family planning interventions is cost effective in a variety of L-MICs as measured against accepted international cost effectiveness benchmarks. In areas with high HIV prevalence, integrating family planning and HIV services can be efficient and cost effective; however the evidence is only supported by a very limited number of studies. The major drivers of cost effectiveness were cost of increasing coverage, effectiveness of the interventions and country-specific factors.

**Conclusion:**

Improving family planning interventions in low and middle income countries appears to be cost-effective. Additional economic evaluation studies with improved reporting quality are necessary to generate further evidence on costs, cost-effectiveness, and affordability, and to support increased funding and investments in family planning programs.

## Introduction

Family planning allows people to attain their desired number of children, which is achieved through the use of effective contraceptive methods [1]. However, despite the decrease of unmet need for family planning globally for the last two decades [2], a significant number of women in low and middle income countries (L-MICs) who need any family planning methods to delay or cease fertility, still experience a lack in access to modern effective methods [1,2]. Ensuring access to family planning services is one of the crucial strategies to ensure the health and well-being of women, as a woman’s abilities to limit, plan and manage her pregnancies have a direct impact on her health outcomes as well as on the outcomes of pregnancies [1].

Unmet need for family planning is associated with a considerable amount of disability-adjusted life years and also one third of maternity-related disease burden [3–5]. It is estimated that by improving family planning interventions, the risk of maternal death can be decreased as much as 40% [3,6]. This risk can be reduced by preventing high-risk pregnancies in for instance, women of high parities, as well as by preventing pregnancies in those who would otherwise be exposed to unsafe abortion [6]. Additionally, unmet need is especially high among adolescents, migrants, urban slum dwellers, refugees, women in the postpartum period and women with HIV [7,8].

Family planning is one of the important drivers of progress towards target of Millennium Development Goal (MDG) no 5, i.e. to improve maternal health [9,10]. Reducing the unmet need for family planning is included in the continuum of care in reproductive, maternal, newborn and child health (RMNCH), which is one of the pillars in MDG 5. Despite the recommendation, access to any of these interventions is still insufficient in many L-MICs [11].

In order to prioritize among many competing global health needs in these resource-constrained regions, evaluation to identify not only effective but also cost-effective strategies needs to be addressed. As a matter of fact, economic evaluation studies to assess both costs and effectiveness of global health interventions are increasingly considered in the decision making process in L-MICs [12]. Some studies have already been published aiming to summarize the evidence on the effectiveness as well as cost effectiveness of approaches to improve maternal and infant health care [13–15]. However, the synthesis of evidence on cost effective strategies in early interventions, such as family planning, remains limited.

The aim of this study is to conduct a systematic review of published economic evaluation studies, providing a synthesis of evidence on costs, consequences and cost-effectiveness of strategies to improve family planning interventions in L-MICs and assess their implication for policy and future research. Increased investments in family planning are needed especially in L-MICs where unmet need is still high [2,16,17]. Additionally, the term ‘unmet need for family planning’ in this study refers to the proportion of women who do not want to become pregnant, but are not using any contraceptive method [8]. Information on the economic value to assess the strategies can contribute to the design of evidence-based, feasible and sustainable policies [12,18].

## Methods

### Literature search

The literature search followed the PRISMA guidelines (Preferred Reporting Items for Systematic Reviews and Meta-Analysis) [19]. Medline (Pubmed), Embase, Popline, The National Bureau of Economic Research (NBER), EBSCOHost, and The Cochrane Library databases were reviewed. In addition, we also searched the homepages of a number of major international organizations which covered research in family planning such as the World Health Organization (WHO), the Guttmacher Institute, the World Bank, United Nations Population Fund (UNFPA), USAID and the Population Council. The combination of terms “AND” and “OR” as well as (MeSH) and text words were used to narrow the search. The key search strategies applied in the databases included several terms related to the following three concepts: 1) family planning, 2) costs or economic evaluation, and 3) L-MICs in accordance with The World Bank (including low income, lower-middle income, and upper-middle income economies) [20]. S1 Appendix summarizes the search terms used in the electronic databases.

### Study selection and inclusion criteria

The initial search results from electronic databases were exported to a reference manager package, i.e. Refworks, and checked for duplicates. Afterwards, preliminary screening based on title and abstract, followed by a full-text review of the selected articles was performed by two reviewers (NZ and ADIvA) using the following inclusion criteria:

- *Type of studies*–Economic evaluation assessing strategies to improve family planning interventions in L-MICs settings (based on The World Bank classification of income groups) [20]. The studies can be in the form of cost-analysis (CA), cost effectiveness analysis (CEA), cost-utility analysis (CUA), and cost-benefit analysis (CBA) [21].
- *Interventions*–All strategies associated with improved family planning interventions (by means of the holistic approach of the program), including interventions to specific population groups with high unmet need such as adolescents, refugees, women in the postpartum period and women with HIV.
- *Participants*–Women in the reproductive age
- *Time limits*–The article search was limited to the period between January 1995 until April 2015.

The articles that were selected from the international organization websites were screened in the same manner. Any disagreements and differences on the study selection were discussed. Economic evaluation studies assessing specific methods of contraceptives, studies exceeding the pre-specified time limits, and conference proceedings were excluded.

### Data extraction

Study characteristics, methodology, study design (including country/setting, perspective, model type, time horizon and discount rates), parameters and results were extracted from full text articles. When several interventions were assessed, only outcomes measured in regard to family planning were extracted. Thresholds based on per capita gross domestic product (GDP) were used for considering cost-effectiveness. When any necessary information was not available in the main text, supplementary data were observed. The funding of a study was directly obtained from acknowledgments or other sources of funding. Extracted information was summarized in Tables 1 and 2.

**Table 1:** Characteristics of the included studies.

**No**	**Study**	**Year**	**Country/Setting**	**Title**	**Countries covered**	**Type of study**	**WHO Region**	**Income level (The World Bank)**	**Funding**
**Improving family planning interventions in L-MICs**									
1	Hu D, et al [25]	2007	Mexico	The Costs, Benefits, and Cost-Effectiveness of Interventions to Reduce Maternal Morbidity and Mortality in Mexico	Single country	CEA & CUA	AMRO	Upper-middle income	John D and Catherine T MacArthur Foundation
2	Goldie SJ, et al [26]	2010	India	Alternative Strategies to Reduce Maternal Mortality in India: A Cost-Effectiveness Analysis	Single country	CEA	SEARO	Lower-middle income	John D and Catherine T MacArthur Foundation
3	Carvalho N, et al [27]	2013	Afghanistan	National and Sub-national Analysis of the Health Benefits and Cost Effectiveness of Strategies to Reduce Maternal Mortality in Afghanistan	Single country	CEA	EMRO	Low-income	John D and Catherine T MacArthur Foundation
4	Erim, et al [28]	2012	Nigeria	Assessing Health and Economic Outcomes of Interventions to Reduce Pregnancy-Related Mortality in Nigeria	Single country	CEA	AFRO	Lower-middle income	John D and Catherine T MacArthur Foundation
5	Babigumira JB, et al [29]	2012	Uganda	Potential Cost-Effectiveness of Universal Access to Modern Contraceptives in Uganda	Single country	CEA & CUA	AFRO	Low-income	William and Flora Hawlett Foundation
6	Kennedy EC, et al [30]	2013	Vanuatu and Solomon Islands	The Case for Investing in Family Planning in the Pacific: Costs and Benefits of Reducing Unmet Need for Contraception in Vanuatu and the Solomon Islands	Multi-country	CEA	WPRO	Lower-middle income	None stated
**No**	**Study**	**Year**	**Country/Setting**	**Title**	**Countries covered**	**Type of study**	**WHO Region**	**Income level (The World Bank)**	**Funding**
**Improving family planning interventions for HIV-positive women in L-MICs**									
7	Reynolds HW, et al [24]	2006	Sub-Saharan Africa	The Value of Contraception to Prevent Perinatal HIV Transmission	Multi-country	CEA	AFRO	Mixed (low-income—upper middle-income)	USAID
8	Halperin DT, et al [31]	2009	14 countries in Africa	Benefits and Costs of Expanding Access to Family Planning Programs to Women Living with HIV	Multi-country	CBA	Multiple	Mixed (low-income—upper middle-income)	USAID
9	Shade SB, et al [32]	2013	Kenya	Cost, Cost-efficiency and Cost-Effectiveness of Integrated Family Planning and HIV Services	Single country	CEA	AFRO	Low-income	The Bill & Melinda Gates Foundation

CEA: Cost effectiveness analysis

CUA: Cost utility analysis

CBA: Cost benefit analysis

AMRO: Regional office for the Americas

SEARO: Regional office for South-East Asia

EMRO: Regional office for the Eastern Mediterranean

AFRO: Regional office for Africa

WPRO: Regional Office for the Western Pacific

USAID: United States Agency for International Development

**Table 2:** Methodological characteristics and results of the included studies.

**No**	**Interventions**		**Comparator**	**Form of economic evaluation**	**Willingness to pay threshold (USD 2014)**	**Time horizon**	**Sensitivity Analysis**	**Outcome measure**	**Result (USD 2014)**	**Quality of reporting**	**Reference**
	**Strategies**	**FP Methods**									
**Improving family planning interventions in L-MICs**											
1	Increasing FP coverage up to 74% (older than 20 years old) and 33% (younger than 20 years old) and improving maternal care	Oral contraceptives, IUD, injectables, condoms, female and male sterilization	Current standard of care (existing coverage of modern FP: 59% in women older than 20 years old, 18% for women younger than 20 years old)	Decision tree	GDP/capita (8,118)	Lifetime	Univariate	ICER per DALY averted	401	Moderate	[25]
2	Stepwise approach to reduce the unmet need by 25%, 50%, 75% until 100%; and improving maternal care	Oral contraceptives, IUD, injectables, condoms, female and male sterilization	Current standard of care (existing coverage of modern FP: 48.5%, unmet need: 13.2%)	Computer-based Global Maternal Health Policy Model*	GDP/capita (1,253)	Lifetime	Univariate (one-way)	ICER per YLS	587	Good	[26]
3	Increasing FP coverage up to 30%-60% and improving maternal care	Oral contraceptives, IUD, injectables, condoms, female and male sterilization, traditional methods	Current standard of care (existing coverage of FP any method: 23%)	Computer-based Global Maternal Health Policy Model*	GDP/capita (587)	Lifetime	Univariate	ICER per YLS	235	Good	[27]
**No**	**Interventions**		**Comparator**	**Form of economic evaluation**	**Willingness to pay threshold (USD 2014)**	**Time horizon**	**Sensitivity Analysis**	**Outcome measure**	**Result (USD 2014)**	**Quality of reporting**	**Reference**
	**Strategies**	**FP Methods**									
**Improving family planning interventions in L-MICs**											
4	Stepwise approach to reduce the unmet need by 25%, 50%, 75% until 100%; and improving maternal care	Oral contraceptives, IUD, injectables, condoms, female and male sterilization	Current standard of care (existing coverage of modern FP: 9.7%, unmet need: 20.2%)	Computer-based Global Maternal Health Policy Model*	GDP/ capita (1,285)	Lifetime	Univariate	ICER per YLS	550	Good	[28]
5	New contraceptive program to reduce the unmet need by 100% (universal access to modern contraceptives)	Oral contraceptives, IUD, injectables, condoms, implants, female and male sterilization	Current contraceptive program (existing coverage of modern FP: 31%)	Markov	GDP/ capita (515)	Lifetime	Univariate & probabilistic	ICER per YLS and per DALY	Dominant**	Good	[29]
6	Two scenarios: 1) all family planning needs (100%) met by 2020; and, 2) all needs met by 2050.	Oral contraceptives, IUD, injectables, condoms, implants, female and male sterilization, traditional methods	No change in unmet need for FP (existing unmet need: Vanuatu 30%; Solomon Islands 11%)	Demographic modelling	NA	5–15 years	Univariate (one-way)	Cost per unintended pregnancy averted	Solomon: 1) 1102) 153 Vanuatu: 1)1142) 154	Moderate	[30]
**No**	**Interventions**		**Comparator**	**Form of economic evaluation**	**Willingness to pay threshold (USD 2014)**	**Time horizon**	**Sensitivity Analysis**	**Outcome measure**	**Result (USD 2014)**	**Quality of reporting**	**Reference**
	**Strategies**	**FP Methods**									
**Improving family planning interventions for HIV-positive women in L-MICs**											
7	Increasing FP coverage to reduce the unmet need by 90%	Oral contraceptives, IUD, injectables, condoms, implants, female and male sterilization	Improved access to HIV testing and counseling, coupled with Nevirapine for PMTCT	Decision tree	NA	1 year	One-way	ICER per HIV-positive birth averted	886	Moderate	[24]
8	Increasing FP coverage to reduce the unmet need by 100%; for women with HIV	Oral contraceptives, IUD, injectables, condoms, implants, female and male sterilization	Current situation (no change in unmet need for FP)	Cost projection	NA	NA	NR	ICER per infant HIV infections averted	390	Low	[31]
9	Integrated family planning services with HIV care and treatment.	Oral contraceptives, IUD, injectables, condoms, implants, female and male sterilization	Standard care of HIV care with separate family planning and HIV services	Trial-based cost effectiveness	NA	1 year	NR	ICER per unintended pregnancy averted	1439	Moderate	[32]

* Identical economic model

** Intervention is less costly and more effective compared to comparator

FP: family planning

IUD: intrauterine devices

NR: Not Reported

NA: Not applicable

GDP: Gross Domestic Product

PMTCT: Prevention of mother to child transmission of HIV

ICER: Incremental cost effectiveness ratio

DALY: Disability adjusted life year

YLS: Year of life saved

### Main findings and data synthesis

The incremental cost effectiveness ratios (ICERs) and other outcome measures were adjusted to 2014 USD by using inflation rates from the World Bank annual Consumer Price Index and purchasing power parities (PPPs) for comparability. In data synthesis, a narrative approach was used to analyze the findings due to diversity in interventions, comparators, methods and study populations. The outcome measures and results from included studies were presented in Table 2.

### Quality of reporting

The quality of reporting of all included studies was assessed using the Consolidated Health Economic Evaluation Reporting Standards (CHEERS) statement [22]. With the intention of obtaining overall reporting quality assessments, studies were assigned 1 point per item if the requirement from the checklist was fulfilled, 0.5 each when partially fulfilled and 0 point when no or insufficient information was reported. Even though the CHEERS checklist is not designed as scoring instrument, the application of a scoring method for CHEERS checklist has been used and published elsewhere [15,23]. Twenty-four checklist items were divided into six main categories (title and abstract, introduction, methods, results, discussion, and other). These items were subsequently calculated as a percentage score with the underlying assumption that all criteria were weighted equally and criteria which were not applicable were excluded from the estimation. Studies with a score higher than 75% were categorized as good, studies in the range 50–74% were categorized moderate and studies with scores lower than 50% were categorized as low. Even though in this way studies will be assigned a quality of reporting score, this score is not a measure for the quality of the study. The mere fact that some items were not reported on does not imply that study quality is low. Therefore, applying the CHEERS checklist was mainly performed to provide additional information and not to generate a weighting factor for study importance.

## Results

**Fig 1:**
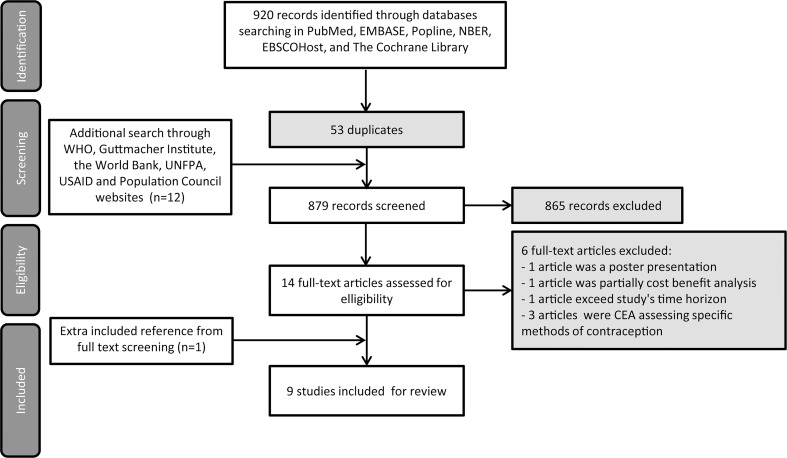
PRISMA flow diagram depicting the process of the study selection.

Fig 1 shows the flow diagram for the identification of studies. The initial database search identified 920 published studies, of which 53 were excluded as duplicates. The additional search on the homepages of international organizations discovered an extra 12 articles which appeared relevant to the topic. The 879 studies thus identified were screened by title and abstract. Based on this screening 865 studies were excluded, mainly because they analyzed a different topic, for instance issues in pregnancy and abortion, concerned non-economic evaluation studies, were not done in L-MICs, or published before the year 1995. The full texts of 14 studies were retrieved for further screening and 6 of these were excluded. One extra relevant study [24] was identified from the included reference during the full text screening, resulting in final inclusion of 9 studies [24–32].

### Overview of included studies

The characteristics of included studies are presented in Table 1. From the included studies, six articles assessed the cost effectiveness of improving family planning interventions in Mexico [25], India [26], Afghanistan [27], Nigeria [28], Uganda [29] and Pacific islands [30], of which four were evaluating family planning as one of several interventions to reduce maternal mortality [25–28]. The remaining three studies assessed strategies to reduce the unmet meet of family planning for HIV-infected women by means of providing integrated family planning and HIV services in multiple countries in Africa [24,31,32].

There were six single-country studies and three multi-country studies. Almost all country-specific studies focused on improving family planning interventions in the general population, while most multi-country studies were in African countries and examined the topic of integrating family planning and HIV services. Moreover, the studies were mainly funded by the private sector/ foundations.

Descriptive information about methodological characteristics and results is presented in Table 2. The healthcare provider perspective was used on most included studies, either mentioned explicitly or not. From all nine studies, six used an economic model (decision tree or Markov), two studies used demographic data and the other one used data from a cluster-randomized trial to perform the evaluation. None of the studies concerning the cost effectiveness of integrated HIV service and family planning used GDP per capita as a benchmark to consider the cost-effectiveness of the interventions. Most other studies however, used GDP per capita as a benchmark, to assess the willingness to pay threshold for their analysis. While most of the studies considered a lifetime time horizon for the analysis, the studies in HIV-positive women considered a relatively short (1 year) time horizon for their analysis. Despite the fact that many studies applied a lifetime time horizon, only very few mentioned or reported the discount rates for both cost and health outcome [26,29,30]. Detailed information about categories of included costs was provided in Table 3.

**Table 3:** Perspective and category of included costs.

Study	Perspective	Cost year and currency	Discount rates		Cost breakdown				
			Cost	Outcomes	Direct costs			Indirect costs	Source of costs
					Family planning costs	Pregnancy-related costs^b^	Other costs		
[25]	NR	USD 2001	NR	NR	Modern contraceptives^a^	Abortion, prenatal care, delivery, preeclampsia/ eclampsia, sepsis obstructed labor, postpartum hemorrhage, postnatal care	Severe anemia, sexually transmitted infection	NA	Secondary data
[26]	NR	USD 2006	3%	NR	Modern contraceptives^a^	Abortion, antenatal care, delivery, community-based interventions, management of complications, postpartum care	Transportation	NA	Secondary data
[27]	NR	USD 2006	NR	NR	Modern & traditional contraceptives^a^	Abortion, antenatal care, delivery, community-based interventions, management of complications, postpartum care	Transportation	NA	Secondary data
[28]	Societal	USD 2008	NR	NR	Modern contraceptives^a^	Abortion, antenatal care, delivery, community-based interventions, management of complications, postpartum care	Transportation	NR	Secondary data
[29]	Societal & governmental	USD 2010	3%	3%	Modern contraceptives^a^ & out of pocket when patients seek for contraceptive services	Antenatal care, miscarriage, induced abortion, ectopic pregnancy, delivery, obstetric hemorrhage, eclampsia	Overhead and capital costs associated with different services	Productivity loss	Secondary data
[30]	Healthcare provider (service delivery)	USD 2010	3%	3%	Modern & traditional contraceptives^a^	NA	Transportation, storage	NA	Secondary data
[24]	Healthcare provider (service delivery)	USD 2000	NA	NA	Modern contraceptives^a^ including distribution, supplies and outreach visits (training, supervision and provider)	NA	PMTCT including drugs, personnel, counseling and testing	NA	Secondary data
[31]	Healthcare provider (service delivery)	Price year not mentioned	NR	NR	Modern contraceptives^a^	NA	Antiretroviral	NA	Secondary data
[32]	NR	USD 2011	NA	NA	Integration of modern contraceptives^a^ and HIV services	NA	Counseling, training	NA	Primary data

IUD = Intrauterine device

PMTCT = Prevention of mother-to-child transmission

^a ^Modern contraceptives including oral contraceptives, injectable contraceptives, barrier contraceptives, intrauterine device, female and male sterilization weighted by the costs of healthcare personnel and services

^b^ Including costs of healthcare personnel and other healthcare materials

#### Improving family planning interventions in L-MICs

This review identified several economic evaluation studies that assessed various strategies to reduce the unmet need for family planning in L-MICs. The results were similar among included studies, suggesting that reducing unmet need for family planning would be highly cost effective, as strategies for spacing and limiting births would have significant benefits for both mothers and infants and also reduce the demand for elective abortion in the regions.

Studies in Mexico [25], India [26], Afghanistan [27] and Nigeria [28] revealed similar results. Increasing the provision of family planning was cost effective and family planning coupled with integrated services for maternal care (such as improved access to antenatal/ postpartum care and emergency obstetrical care) would prevent four out of five maternal deaths and have an ICER less than countries with an equal GDP/ capita per year live saved (YLS), a common benchmark for cost effectiveness. Compared to other strategies to reduce maternal morbidity and mortality, reducing the unmet need for family planning is the most cost-effective single intervention. These four studies used a similar decision analytic model to assess the potential cost effectiveness of the various strategies and extrapolated the model to each country’s settings. While the model estimated the natural course of pregnancy using a lifetime time horizon, most studies did not estimate both discounted cost and health outcomes. All studies performed similar deterministic sensitivity analyses with rather diverse results. With regard to family planning, it seemed that varying the cost of increasing coverage and also effectiveness of the interventions had the highest impact on the outcomes [25–28].

In Uganda [29], reducing the unmet need and improving the universal access to modern contraception appeared to be highly cost effective compared to the status quo where the access was limited, as seen from both a societal and governmental perspective. The model compared the new strategy with the current situation with regard to costs, life expectancy, ICER per life year saved (LYS) and disability-adjusted life years (DALY) averted during a lifetime time horizon. The analyses suggested that mean discounted life expectancy was slightly higher for the new strategy with ICER/ DALY dominating the comparator, i.e. the new strategy would be less costly at a more favorable health outcome. Univariate and probabilistic sensitivity analyses were performed to assess which parameters had significant impact on results. The results showed that cost of both contraception and pregnancy appeared to be the most sensitive variables for incremental cost and discount rate was the most sensitive parameter for incremental DALYs. It was also uncertain whether the new strategy was less costly than the current strategy. The new strategy took into account not only the cost of the interventions but also any costs on the consequences, as well as the associated costs or savings further down the line. However, it was very likely that the new strategy was more effective, as quantified in probabilistic sensitivity analysis.

The projected health benefits associated with an increased investment to reduce the unmet need for family planning were modeled by Kennedy, et al for Vanuatu and the Solomon islands, small developing states in the Pacific Islands [30]. Demographic modeling was used in the analysis to assess the economic consequences of the strategy. It was estimated that meeting the needs of family planning would increase modern contraceptive use approximately by 20% for both states and reduce the rate of unintended pregnancy and high-risk births as well as the number of abortions significantly. This strategy would also slow down annual population growth, and therefore lower the youth dependency ratio. The analysis took a service delivery perspective, and included all direct costs associated with providing family planning. Increased investment in family planning in these small states was estimated to result in considerable public sector savings [30].

#### Improving family planning interventions for HIV-positive women in L-MICs

The remaining three studies in the review assessed the impact of improved family planning interventions in HIV-positive women, mainly to prevent unintended pregnancy among HIV-positive women who do not wish to become or want to delay pregnancy and to avert HIV transmission from mothers to their infants. The overall results from all studies suggested that integrated family planning and HIV services were considered cost-effective and efficient. Sensitivity analysis to assess the uncertainty that may be present in the results was not performed in most of these studies, making it difficult to evaluate reliability of the conclusions.

A study by Reynolds et al evaluated cost effectiveness of family planning to prevent unintended pregnancy among HIV-positive women in sub-Saharan Africa [24]. Evaluation of perinatal HIV transmission prevention in a hypothetical sub-Saharan Africa cohort was modeled to assess relative cost-effectiveness of universal access to family planning to reduce the unmet need by approximately 90% for all sexually active women, compared with improved access to HIV testing and counseling coupled with nevirapine prophylaxis (referred to nevirapine for PMTCT in prenatal care) to avert HIV-positive births. The results suggested that reducing the unmet need of family planning without consideration of HIV status is at least as cost-effective as reducing HIV transmission by nevirapine for PMCTC. The study estimated that increasing contraceptive use among all women who do not wish to become pregnant would avert approximately 30% more HIV-positive births than the other strategy would prevent [24]. Sensitivity analysis on different assumptions about the costs of either programs or effectiveness of nevirapine for PMTCT suggested that effectiveness of nevirapine seemed to be the most sensitive parameter in the model.

Cost effectiveness of integrated family planning and HIV services was the main topic of two studies by Halperin, et al [31] and Shade, et al [32]. Both studies suggested that the approach to integrate family planning and HIV services was efficient as well as cost-effective to prevent perinatal HIV transmission and unintended pregnancies [31,32]. Family planning becomes an important aspect in HIV services, as women and couples with HIV may access family planning not only to prevent unintended pregnancy but also to plan healthy pregnancy when desired [33]. Halperin et al estimated that providing antiretroviral (ARV) prophylaxis would prevent approximately two hundred thousand HIV infections to infants in countries with high-level epidemics of HIV. However, an estimated seventy thousand infants would still be infected with HIV, even with full access of ARV prophylaxis in prenatal care. These infections could have been averted by preventing unintended pregnancy via family planning.

Meanwhile, analysis of a cluster-randomize trial in one province in Kenya [32] estimated that integration of family planning in HIV services was feasible, efficient and also cost effective in the Kenyan setting with the costs being within the range of the estimated value and higher efficacy than the previous estimation taken from the study by Reynolds et al [24].

### Quality of reporting

Based on reporting quality assessment from the CHEERS checklist, four studies were ranked as good, four as moderate and the other one was categorized as low. Studies which focused on expanding family planning interventions were rated as having good or moderate quality of reporting; studies which focused on integrating family planning into HIV care were rated as having moderate or low quality of reporting.

Table 4 shows the proportion of each item in the CHEERS checklist that is reported sufficiently, partially, or not at all by all included studies in the review. Most studies failed to fully report details on how resources and costs were collected and estimated, along with poor reporting of the discount rate. Additionally, four out of nine studies also did not report study perspective when describing included costs. Most studies reported the incremental cost effectiveness ratio of the family planning interventions compared to alternative strategies or current situation. Discussions related to key findings, limitations and generalizability mostly provided although several studies did not comprehensively assess these criteria. Only half of studies reported source of funding and potential conflicts of interest. Furthermore, no studies stated the role of funder in the identification, design, conduct and reporting of the analysis.

**Table 4:** CHEERS checklist per item for all included studies in the review.

CHEERS section/item	Item No	References								
		[25]	[26]	[27]	[28]	[29]	[30]	[24]	[31]	[32]
**Title and abstract**										
Title	1	Y	Y	Y	Y	Y	Y	N	Y	Y
Abstract	2	Y	Y	Y	Y	Y	Y	P	P	P
**Introduction**										
Background and objectives	3	Y	Y	Y	Y	Y	Y	P	Y	Y
**Methods**										
Target population and subgroups	4	Y	Y	Y	Y	Y	Y	Y	Y	Y
Setting and location	5	Y	Y	Y	Y	Y	Y	Y	Y	Y
Study perspective	6	N	N	N	Y	Y	Y	Y	Y	N
Comparators	7	Y	Y	Y	Y	Y	Y	P	NA	Y
Time horizon	8	Y	Y	Y	Y	Y	P	Y	N	N
Discount rate	9	N	P	N	N	Y	Y	NA	N	N
Choice of health outcomes	10	Y	Y	Y	Y	Y	P	Y	P	P
Measurement of effectiveness (single study-based estimates)	11a	NA	NA	NA	NA	NA	NA	NA	N	P
Measurement of effectiveness (synthesis-based estimates)	11b	P	P	P	P	Y	P	P	P	NA
Measurement and valuation of preference based outcomes	12	Y	Y	Y	Y	Y	NA	N	NA	P
Estimating resources and costs (single study-based economic evaluation)	13a	NA	NA	NA	NA	NA	NA	NA	NA	P
Estimating resources and costs (model-based economic evaluation)	13b	P	P	P	P	P	Y	P	P	NA
Currency, price date, and conversion	14	Y	Y	Y	Y	Y	Y	Y	N	Y
Choice of model	15	Y	Y	Y	Y	Y	P	P	N	NA
Assumptions	16	Y	Y	Y	Y	Y	Y	Y	N	N
Analytical methods	17	P	P	P	P	P	P	P	P	P
**Results**										
Study parameters	18	Y	Y	Y	Y	Y	P	Y	P	Y
Incremental costs and outcomes	19	Y	Y	Y	Y	Y	P	Y	P	Y
Characterizing uncertainty (single study-based economic evaluation)	20a	NA	NA	NA	NA	NA	NA	NA	NA	N
Characterizing uncertainty (model-based economic evaluation)	20b	P	Y	P	Y	Y	P	Y	N	NA
Characterising heterogeneity	21	NA	NA	NA	NA	NA	NA	NA	NA	N
**Discussions**										
Study findings, limitations, generalizability, and current knowledge	22	Y	Y	Y	Y	Y	P	P	P	P
**Other**										
Source of funding	23	N	N	Y	Y	Y	N	Y	N	Y
Conflict of interest	24	N	N	Y	Y	Y	N	N	Y	Y
Reporting quality based on % score*		Moderate	Good	Good	Good	Good	Moderate	Moderate	Low	Moderate

Yes: reported, Part: partially reported, No: not reported, NA: not applicable

*Studies were assigned 1 point per item for Yes, 0.5 for part, and 0 for No. Percentage score was calculated after the exclusion of “not applicable” item

## Discussion

We conducted a systematic review of economic evaluation studies assessing strategies to improve family planning interventions by reducing the unmet need in resource-limited settings. Generally, the included studies suggested that the interventions to increase the prevalence of family planning in a variety of L-MIC settings can be cost-effective or cost saving as measured against accepted thresholds for estimating cost effectiveness [34]. There was diversity in the strategies, the measures of effectiveness and outcomes, the scales of implementation and also the study setting. In addition, the number of studies in this area is still limited, which limits our ability to draw strong conclusions.

This review also highlighted the lack of economic evaluation studies that assessed explicit interventions to improve both supply and demand for contraceptives in L-MICs. During the 1970s-1980s, family planning programs were on the rise with increased funding from international support, and it was considered an essential period since important developments such as increased use of contraceptives and reduced fertility were observed all over the world [10,35], A more comprehensive rationale for family planning programs was also introduced after this period, with the purpose of women’s empowerment and reproductive health and rights [10]. However, family planning has shifted away from international development priorities that led to limited funding in the period from the 1990s [10,35], up until recently when increased attention and renewed focus on family planning were being re-introduced [1,36,37]. Over the last years, broadening the discussion regarding the impact of family planning on the socio-economic development and demographic dividend, in addition to health and rights of women and girls, has contributed to this renewed and enhanced focus [10]. With high unmet need and relatively low modern contraceptives coverage in LMICs, information on costs and cost-effectiveness of investments in strategies to reduce the unmet need seems needed for policymakers in order to effectively allocate limited resources.

The conclusion from the studies which assessed family planning as a possible strategy to reduce maternal morbidity and mortality, suggested that these early intensive efforts to scale up family planning interventions contributed significantly to the decreasing number of maternal deaths [25–28]. Compared to other interventions that were assessed in addition to increased family planning i.e safe abortion, increased skilled attendants, improved antenatal/postpartum care, incrementally shifted births away from home, and improved availability and quality of emergency obstetrical care (EmOC), family planning was the most cost-effective individual intervention to reduce maternal mortality. The implementation of this early intervention seemed to play an important role since strategies to improve contraceptive options as a way to assist fertility choices for women do not require complex integrated infrastructure as opposed to the other strategies [25–28].

Women living with HIV are a population with a considerable unmet need for family planning [38,39], thus averting unintended pregnancy among this population is a cost-effective way to improve maternal and child health [40]. Concerns in the global health community had shifted during the past ten years from reproductive health to HIV [39]. However, recently it has been suggested that integrating HIV with reproductive health has potential to improve the quality, continuity and efficiency of care for those living with HIV [41]. There were three studies in this review that analyzed the cost effectiveness of family planning for HIV-positive women, all of them performed in African countries. The vast majority of people living with HIV are in the low and middle income countries, with sub-Saharan Africa as the most affected region [42]. The conclusions from most of the included studies were in line with current WHO recommendations on integration of HIV services. However, support for integration of family planning into HIV care is only supported by a very limited number of studies with low to moderate quality of reporting. Therefore, additional studies are needed to better describe the potential economic benefits of integrated HIV care and family planning services.

Favorable health consequences with regard to family planning can contribute to achieve some points in MDG 5, mainly by reducing the number of unintended pregnancies as well as maternal and infant morbidity and mortality [17]. Investment in women’s and children’s health would also substantially secure health, economic and social returns. It was suggested that scaling up access to contraceptives would be a predominantly cost-effective investment that contributes to the prevention of maternal death [17].

Studies that reported the outcomes in a measure of cost per year life saved (YLS) or cost per disability adjusted life year (DALY) averted concluded that the strategies involving increased access of family planning in some of developing countries were cost-effective when compared to their GDP per capita [25–29]. DALY is a widely accepted health impact metric for cross-country comparisons and the outcomes of the cost per DALY averted is widely accepted as a benchmark to assess the cost utility of healthcare interventions and has been widely used to directly compare relative cost-effectiveness in different national settings [43]. However, using GDP per capita as a threshold to estimate the cost-effectiveness of interventions might only be valid from a national perspective and is therefore not applicable outside the study’s setting [44,45]. Also, using national thresholds when making international comparisons is somewhat risky, mainly because it does not distinguish cost-effectiveness and affordability [44]. The alternative approaches for estimating thresholds for cost effectiveness are using benchmarks interventions or league tables, if possible [45]. The threshold for benchmark interventions is established by retrospective analysis of relevant current practice, while league tables is to basically rank all relevant options according to their ICERs. The purpose for these methods is to facilitate decision makers with more thorough assessment on relevant alternatives to efficiently utilize national welfare [45].

In global health issues, where the funding could also come from non-governmental organizations, the cost-effectiveness analysis from a transnational perspective with equal value gains would allow for a cross country comparison and enforce resource allocation that would maximize health gains [44].

A lack of empirical data on both costs and effectiveness, especially for approaches to increase supply and demand for modern contraceptives, was observed among included studies, which indicates a need for trials in this particular area. Despite the many advantages a modeling framework offers, such as the synthesis of evidence from multiple sources, enhanced with relevant assumptions to extrapolate the outcomes over a longer time horizon [46], empirical data remains essential as a steady basis for these decision analytic models and to reduce uncertainty in the model outcomes.

The CHEERS checklist provides standards for the kind of information that should be reported in economic evaluation studies. Even though there were studies that have adequately fulfilled the standards in the checklist, many studies did not comply with the guidelines. In some studies, inadequacy in reporting made it difficult to actually assess whether the methodology used in the analysis, such as the approach to estimate resources and costs, the costing perspective, the time horizon, discount rates and analytical methods was appropriate. Some studies provided sufficient information in the appendices, which can be an alternative, as many peer-reviewed journals have restrictions on the length of an article.

To the best of our knowledge, this study is one of the first reviews assessing the health economics of strategies to improve family planning interventions in L-MICs [47]. Previous studies that evaluated cost effectiveness of strategies to improve maternal and infant health suggested that improving the utilization and provision of maternal and infant healthcare is cost effective in low income and lower-middle income countries [15]. However, provision of family planning as the early intervention in RMNCH had not been assessed exclusively until now. Part of the result from this review also elaborated on previous review, which assessed the costs and efficiency of integrating HIV/AIDS services with other health services [41]. It was concluded that a range of integrated HIV services were found to be cost effective, however the evidence to integrate HIV services with family planning services remain limited [41].

The results of this review are of importance to decision makers, as the results of cost-effectiveness analyses should be compared with many other relevant interventions in a specific context. Therefore decision makers would be in a better position to interpret and prioritize among the many competing global health needs.

Inevitably, this review has several limitations. We took a systematic approach in the literature search and screening process; however there is a chance that there were relevant studies that have been missed. In addition, due to variability of interventions and strategies that were assessed in the included studies, comparability of studies is limited. However in this review, we facilitate this with a narrative approach, therefore the variation in methodology and study designs can be observed thoroughly. Also, as all the included studies in this review had positive findings, therefore publication bias is also a potential limitation. This could mean that cost effectiveness analyses with negative findings might not have been published. Individual interpretation while assessing the quality of reporting could also lead to bias as the checklist sometimes contained several recommendations per point.

## Conclusion

This review provides a comprehensive health economic assessment of the available published studies of improving family planning interventions in L-MICs. Due to increased attention for economic evaluation of healthcare interventions in these countries, the results of this review can be essential for any decision maker at different levels to assess the cost-effectiveness of improving global health interventions, especially family planning.

In conclusion, improving family planning interventions to decrease the unmet need in low and middle income countries appears to be cost-effective, however it depends on each country’s thresholds for considering cost effectiveness. Additional economic evaluation studies with improved reporting quality are necessary to generate further evidence on costs, cost-effectiveness, and affordability, and to support increased funding and investments in family planning programs.

## Supporting Information

S1 Appendix Full search strategy. (DOCX)

S1 Checklist PRISMA checklist. (DOC)

S2 Checklist CHEERS checklist. (DOCX)
