# Supplementary material for: Economic Evaluation of Family Planning Interventions in Low and Middle Income Countries; A Systematic Review
Source: PLoS One. 2016 Dec 19;11(12):e0168447. doi: 10.1371/journal.pone.0168447 (PMC5167385; doi:10.1371/journal.pone.0168447)
Supplement: S1 Appendix — (DOCX) [file pone.0168447.s001.docx]

**Appendix S1**

**Search strategy**

PUBMED : “AND” combination of

*Family planning terms:*

(("Family Planning Services"[Mesh] OR "Family Planning Services"[tw] OR Family Planning Services) OR ("Contraception"[Mesh] OR "Contraceptive Agents"[Mesh] OR contracept*[tw]) AND ("Pregnancy, Unplanned"[Mesh] OR pregnan*[tw]))

*Cost terms:*

("costs and cost analysis"[MeSH Terms] OR "Cost-Benefit Analysis"[Mesh] OR "economics" [Subheading] OR cost[tw]OR costs[tw] OR cost of illness[tw] OR cost-utility[tw] OR cost-effectiveness OR economic eval*[tw]

*Countries terms (LI/MI countries according to The World Bank):*

(("Developing Countries"[Mesh] OR developing[tw] OR "Africa"[Mesh] OR "Asia, Southeastern"[Mesh] OR "Asia, Central"[Mesh] OR "Asia, Western"[Mesh] OR "Mongolia"[Mesh] OR "Caribbean Region"[Mesh] OR "Central America"[Mesh] OR "South America"[Mesh] OR "Transcaucasia"[Mesh] OR "Europe, Eastern"[Mesh] OR "Pacific Islands"[Mesh] OR Afghan*[tw] OR Guinea*[tw] OR Peru*[tw] OR alban*[tw] OR Philippin*[tw] OR Algeria*[tw] OR Haiti*[tw] OR Romania*[tw] OR Samoa*[tw] OR Hondura*[tw] OR Rwand*[tw] OR Angola*[tw] OR Hungar*[tw] OR Argentin*[tw] OR India[tw] OR São Tomé[tw] OR Armeni*[tw] OR Indonesia*[tw] OR Senegal*[tw] OR Azerbaijan*[tw] OR Iran*[tw] OR serbia*[tw] OR Banglad*[tw] OR Iraq*[tw] OR Seychell*[tw] OR Belarus*[tw] OR Jamaic*[tw] OR Sierra Leon*[tw] OR Beliz*[tw] OR Jordan*[tw] OR Solomon[tw] OR Benin*[tw] OR Kazakhstan* OR Somalia*[tw] OR Bhutan*[tw] OR Kenya*[tw] OR South Africa*[tw] OR Bolivia*[tw] OR Kiribati[tw] OR South Sudan*[tw] OR Bosnia*[tw] OR Herzegovin*[tw] OR Korea*[tw] OR Sri Lank*[tw] OR Botswan*[tw] OR Kosovo[tw] OR St. Lucia[tw] OR Brazil*[tw] OR Kyrgyz*[tw] OR St. Vincent[tw] OR Grenadin*[tw] OR Bulgaria*[tw] OR Sudan*[tw] OR Burkina Faso*[tw] OR Leban*[tw] OR Surinam*[tw] OR Burund*[tw] OR Lesoth*[tw] OR Swaziland*[tw] OR Cabo Verd*[tw] OR Liberia*[tw] OR Syria*[tw] OR Cambodia*[tw] OR Libya*[tw] OR Tajikistan*[tw] OR Cameroon*[tw] OR Macedonia*[tw] OR Tanzania*[tw] OR Central African Republic[tw] OR Madagascar[tw] OR Thailand[tw] OR Chad[tw] OR Malaw*[tw] OR Timor[tw] OR Chin*[tw] OR Malaysia*[tw] OR Togo[tw] OR Colombia*[tw] OR Maldives[tw] OR Tonga[tw] OR Comoros[tw] OR Mali[tw] OR Tunis*[tw] OR Congo*[tw] OR Marshall[tw] OR Turk*[tw] OR Maurit*[tw] OR Costa Ric*[tw] OR Tuvalu*[tw] OR Côte d'Ivoire[tw] OR ivory coast[tw] OR Mexic*[tw] OR Ugand*[tw] OR Cuba*[tw] OR Micronesia*[tw] OR Ukrain*[tw] OR Djibouti*[tw] OR Moldov*[tw] OR Uzbek*[tw] OR Mongolia*[tw] OR Vanuatu*[tw] OR Dominica*[tw] OR montenegr*[tw] OR Venezuela*[tw] OR Ecuador*[tw] OR Morocc*[tw] OR Vietnam*[tw] OR Egypt*[tw] OR Arab[tw] OR arabia*[tw] OR Mozambi*[tw] OR west Bank[tw] OR Gaza[tw] OR El Salvador*[tw] OR Myanmar[tw] OR Yemen*[tw] OR Eritrea*[tw] OR Namibia*[tw] OR Zambia*[tw] OR Ethiopia[tw] OR Nepal*[tw] OR Zimbabw*[tw] OR Fiji[tw] OR Nicara*[tw] OR Gabon*[tw] OR Niger*[tw]))

EMBASE

'family planning'/exp OR 'family planning' OR 'contraception'/exp OR 'contraception' OR 'contraceptive agents'/exp OR 'contraceptive agents' OR 'contraceptive'/exp OR 'contraceptive' AND ('pregnancy, unplanned'/exp OR 'pregnancy, unplanned' OR pregnan*) AND ('costs and cost analysis'/exp OR 'costs and cost analysis' OR 'cost-benefit analysis'/exp OR 'cost-benefit analysis' OR costs OR 'cost'/exp OR cost ANDof AND ('illness'/exp OR illness) OR 'cost utility'/exp OR 'cost utility' OR 'cost effectiveness'/exp OR 'cost effectiveness' OR economic) AND eval* AND ('developing countries'/exp OR 'developing countries' ORdeveloping OR 'africa'/exp OR 'africa' OR 'asia, southeastern'/exp OR 'asia, southeastern' OR 'asia, central'/exp OR 'asia, central' OR 'asia, western'/exp OR 'asia, western' OR 'mongolia'/exp OR 'mongolia' OR'caribbean region'/exp OR 'caribbean region' OR 'central america'/exp OR 'central america' OR 'south america'/exp OR 'south america' OR 'transcaucasia'/exp OR 'transcaucasia' OR 'europe, eastern'/exp OR 'europe, eastern' OR 'pacific islands'/exp OR 'pacific islands')

POPLINE

(family planning) AND (contraceptives) AND (unintended pregnan* OR unwanted pregnan*) AND (cost of illness OR cost analysis OR cost effectiveness OR cost benefit OR cost utility OR economic evaluation OR economic analysis OR health economics) AND

("Developing Countries" OR developing OR "Africa" OR "Asia, Southeastern" OR "Asia, Central" OR "Asia, Western" OR "Mongolia" OR "Caribbean Region" OR "Central America" OR "South America" OR "Transcaucasia" OR "Europe, Eastern" OR "Pacific Islands" OR "Afghan*" OR "Guinea*" OR "Peru*" OR "alban*" OR "Philippin*" OR "Algeria*" OR "Haiti*" OR "Romania*" OR "Samoa*" OR "Hondura*" OR "Rwand*" OR "Angola*" OR "Hungar*" OR "Argentin*" OR "India" OR "São Tomé" OR "Armeni*" OR "Indonesia*" OR "Senegal*" OR "azerbaijan" OR "Iran*" OR "serbia*" OR "Banglad*" OR "Iraq*" OR "Seychell*" OR "Belarus*" OR "Jamaic*" OR "Sierra Leon*" OR "Beliz*" OR "Jordan*" OR "Solomon" OR "Benin*" OR "Kazakhstan*" OR "Somalia*" OR "Bhutan*" OR "Kenya*" OR "South Africa*" OR "Bolivia*" OR "Kiribati" OR "South Sudan*" OR "Bosnia*" OR "Herzegovin*" OR "Korea*" OR "Sri Lank*" OR "Botswan*" OR "Kosovo" OR "St. Lucia" OR "Brazil*" OR "Kyrgyz*" OR "St. Vincent" OR "Grenadin*" OR "Bulgaria*" OR "Sudan*" OR "Burkina Faso*" OR "Leban*" OR "Surinam*" OR "Burund*" OR "Lesoth*" OR "Swaziland*" OR "Cabo Verd*" OR "Liberia*" OR "Syria*" OR "Cambodia*" OR "Libya*" OR "Tajikistan*" OR "Cameroon*" OR "Macedonia*" OR "Tanzania*" OR "Central African Republic" OR "Madagascar" OR "Thailand" OR "Chad" OR "Malaw*" OR "Timor*" OR "Chin*" OR "Malaysia*" OR "Togo" OR "Colombia*" OR "Maldives" OR "Comoros" OR "Mali" OR "Tunis*" OR "Congo*" OR "Marshall" OR "Turk*" OR "Maurit*" OR "Costa Ric*" OR "Tuvalu*" OR "Côte d'Ivoire" OR "ivory coast" OR "Mexic*" OR "Ugand*" OR "Cuba*" OR "Micronesia*" OR "Ukrain*" OR "Djibouti*" OR "Moldov*" OR "Uzbek*" OR "Mongolia*" OR "Vanuatu*" OR "Dominica*" OR "montenegr*" OR "Venezuela*" OR "Ecuador*" OR "Morocc*" OR "Vietnam*" OR "Egypt*" OR "Arab" OR "arabia*" OR "Mozambi*" OR "west Bank" OR "Gaza" OR "El Salvador*" OR "Myanmar" OR "Yemen*" OR "Eritrea*" OR "Namibia*" OR "Zambia*" OR "Ethiopia" OR "Nepal*" OR "Zimbabw*" OR "Fiji" OR "Nicara*" OR "Gabon*" OR "Niger*")

THE COCHRANE LIBRARY, limited economic evaluation

((family planning OR contraceptives) AND (unintended pregnancy OR unwanted pregnancy) AND (cost of illness OR cost analysis OR cost effectiveness OR cost benefit OR cost utility OR economic evaluation OR economic analysis OR health economics))

EBSCOHost/ EconLit

(Reproductive health OR Sexual health) AND (family planning) AND (contraceptives) AND (unintended pregnan* OR unwanted pregnan*) AND (cost of illness OR cost analysis OR cost effectiveness OR cost benefit OR cost utility OR economic evaluation OR economic analysis OR budget impact OR health economics)

National Bureau of Economic Research (NBER)

Reproductive health family planning contraceptives unintended pregnancy economic evaluation cost effectiveness
